# Microstructures and Rheological Properties of Short-Side-Chain Perfluorosulfonic Acid in Water/2-Propanol

**DOI:** 10.3390/polym16131863

**Published:** 2024-06-29

**Authors:** Yan Qiu, Xinyang Zhao, Hong Li, Sijun Liu, Wei Yu

**Affiliations:** 1 Advanced Rheology Institute, Department of Polymer Science and Engineering, Shanghai Jiao Tong University, Shanghai 200240, China; 2 Shanghai Electrochemical Energy Devices Research Center, School of Chemistry and Chemical Engineering, Shanghai Jiao Tong University, Shanghai 200240, China

**Keywords:** short-side-chain perfluorosulfonic acid, microstructures, rheological scaling, diffusing wave spectroscopy, viscoelasticity

## Abstract

The viscosity and viscoelasticity of polyelectrolyte solutions with a single electrostatic interaction have been carefully studied experimentally and theoretically. Despite some theoretical models describe experimental results well, the influence of multiple interactions (electrostatic and hydrophobic) on rheological scaling is not yet fully resolved. Herein, we systematically study the microstructures and rheological properties of short-side-chain perfluorosulfonic acid (S-PFSA), the most promising candidate of a proton exchange membrane composed of a hydrophobic backbone with hydrophilic side-chains, in water/2-propanol. Small-angle X-ray scattering confirms that semiflexible S-PFSA colloidal particles with a length of ~38 nm and a diameter of 1–1.3 nm are formed, and the concentration dependence of the correlation length (ξ) obeys the power law ξ~c^−0.5^ consistent with the prediction of Dobrynin et al. By combining macrorheology with diffusing wave spectroscopy microrheology, the semidilute unentangled, semidilute entangled, and concentrated regimes corresponding to the scaling relationships η_sp_~c^0.5^, η_sp_~c^1.5^, and η_sp_~c^4.1^ are determined. The linear viscoelasticity indicates that the entanglement concentration (c_e_) obtained from the dependence of η_sp_ on the polymer concentration is underestimated owing to hydrophobic interaction. The true entanglement concentration (c_te_) is obtained by extrapolating the plateau modulus (G_e_) to the terminal modulus (G_t_). Furthermore, G_e_ and the plateau width, τ_r_/τ_e_ (τ_r_ and τ_e_ denote reptation time and Rouse time), scale as G_e_~c^2.4^ and τ_r_/τ_e_~c^4.2^, suggesting that S-PFSA dispersions behave like neutral polymer solutions in the concentrated regime. This work provides mechanistic insight into the rheological behavior of an S-PFSA dispersion, enabling quantitative control over the flow properties in the process of solution coating.

## 1. Introduction

A polyelectrolyte is a type of polymer that possesses many ionizable groups [1,2,3,4]. The electrostatic forces between ionized groups, as dissolved in a polar solvent like water, promote solution stability and result in expanded chain conformations. For polyelectrolyte solutions with a single electrostatic interaction, microstructures, such as contour length and correlation size, and rheological properties, such as specific viscosity and terminal relaxation time, have been studied exhaustively, and some universal scaling laws have been established [5,6,7,8]. For example, the solution viscosity obeys the well-known Fuoss law, and the relaxation time agrees with scaling prediction in the semidilute unentangled regime [9]. However, many studies indicate that the hydrophobic interaction on polymer chains promotes transient intra- or intermolecular association, further resulting in complex microstructures and rheological performances [10,11]. For polyelectrolytes consisting of a hydrophilic backbone and hydrophobic side chains, such as alkyl-chain-grafted chitosan [12], the intra- or intermolecular interaction among the side chains produces hydrophobic microdomains. In a highly dilute aqueous solution, intramolecular hydrophobic interaction may be favorable. However, intermolecular hydrophobic interaction tends to occur as the polymer concentration increases. Polyelectrolyte solutions with intramolecular hydrophobic interactions may have polymer micelles composed of individual macromolecular chains, yielding a much less viscous solution even at a high polymer concentration. In contrast, polyelectrolytes with a strong propensity for intermolecular hydrophobic interaction may cause a large increase in the solution viscosity with an increase in polymer concentration, which may be followed by gelation upon further increasing the polymer concentration. For polyelectrolytes composed of a hydrophobic backbone and a hydrophilic side chain terminated with charged groups, the microstructure and rheological properties of the polyelectrolyte solution are governed by the competition between two opposite effects: attractive association of hydrophobic backbones and electrostatic repulsion between side-chain charged groups. The regularity of the side chains has an important influence on the hydrophobic association of the backbone [13]. Backbones with regularly grafted side chains usually associate into rod-like particles surrounded by a hydrophilic surface, yielding a weak increase in the viscosity. However, non-uniform side chains usually produce unsubstituted regions in the resultant associated rod-like particles, which act as temporary cross-links causing a marked increase in the viscosity [14,15]. A few molecular simulations were performed to confirm these multiple interactions [16,17]. But there are limited experimental data to explore the effect of hydrophobic interaction on scaling laws and viscoelasticity [18]. It is fundamental to understand the microstructures and rheological properties of polyelectrolytes with multiple interactions for industrial application and fundamental polymer science development.

Short-side-chain perfluorosulfonic acid (S-PFSA) is composed of a hydrophobic poly(tetrafluoroethylene) backbone with two carbon perfluoropolyether hydrophilic side chains terminated by sulfonic groups [19]. The amphiphilic properties of S-PFSA promote nanoscale phase separation between the hydrophobic backbone and the hydrophilic side chains [20]. Therefore, the dissociated protons from the sulfonic acid moieties can be transferred through the phase-separated hydrophilic domains. Owing to this unique structure-driven multifunctionality, an S-PFSA membrane, functioning as an insulator to drive electrons through an external circuit and at the same time as a separator of reactant gases, is regarded as the heart of fuel cells [21,22,23,24]. An important method for the preparation of S-PFSA membranes is the so-called solution coating, since the coating technology produces membranes with superior uniformity and enhanced mechanical properties [25,26,27]. Therefore, understanding the rheological properties of S-PFSA dispersions is crucial to the success of the coating process and also determines the physical property and functional performance of the resulting membrane.

Herein, we systematically study the microstructure and rheological properties of S-PFSA in water/2-propanol through small-angle X-ray scattering (SAXS), macrorheology, and diffusing wave spectroscopy (DWS) microrheology. SAXS confirms that the colloidal particles are formed. The correlation length (interparticle distance), ξ, obeys the relationship ξ~c^−0.5^, which is consistent with the scaling prediction. The linear viscoelasticity indicates that the entanglement concentration (c_e_) determined from the dependence of η_sp_ is underestimated. The true entanglement concentration (c_te_) can be obtained by extrapolating the plateau modulus (G_e_) to the terminal modulus (G_t_). Similar rheological performances were also observed for S-PFSA dispersions in water/ethanol and long-side-chain perfluorosulfonic acid (L-PFSA) dispersions in water/2-propanol. These results indicate that polyelectrolyte solutions with multiple interactions demonstrate complex rheological properties.

## 2. Experimental Section

### 2.1. Materials

S-PFSA and L-PFSA powders were provided by Shandong Dongyue Polymer Materials Co., Ltd. (Zibo, China) and were further confirmed by nuclear magnetic fluorine spectrum and FT-IR (Figures S1 and S2, Supplementary Materials). Based on the information provided by the manufacturer, the EW (equivalent weight, the grams of S-PFSA resin corresponding to 1 mole sulfonic acid) and the IEC (ion exchange capacity, the millimoles of sulfonic acid contained in each gram of S-PFSA resin) of S-PFSA were 700 g/mol and 1.43 mol/g, respectively. The EW and IEC of L-PFSA were 910 g/mol and 1.1 mol/g, respectively. Both S-PFSA and L-PFSA were purified and freeze-dried before delivery. 2-Propanol and ethanol (reagent grade) were purchased from Aladdin Chemistry Co., Ltd. (Shanghai, China). Ultra-pure quality water was produced using a PURELAB UHQ II-ELGA Lab Water setup.

### 2.2. Preparation of Dispersions

The dispersions of S-PFSA were fabricated based on two approaches. For concentrations of S-PFSA above 100 mg/mL, the S-PFSA powder was dispersed in water/2-propanol or water/ethanol (50/50 vol/vol) and then vigorously stirred under 60 °C for 24 h, resulting in clear dispersions. For S-PFSA concentrations below 100 mg/mL, the dispersions were prepared by directly diluting the S-PFSA dispersion of 100 mg/mL into the desired concentrations. The L-PFSA dispersions were fabricated following the same procedure. The prepared dispersions were cooled and then stored at ambient temperature before all measurements. And the schematic diagram of the dispersion preparation is shown in Figure S3.

### 2.3. Macrorheology

Macroscopic rheological measurements of the dispersions were performed using a rotational rheometer (Thermo Haake, Inc., Rheinfelden, Germany) under the stress-controlled mode at 25 °C, with the parallel plate geometry in a diameter of 60 mm and a gap of 0.5 mm. The samples for rheological measurement were transferred directly from a glass bottle to the rheometer using a pipette. To prevent the evaporation of the solvent during rheological measurements, a thin layer of low-viscosity silicone oil was placed on the peripheral surface of dispersions held between plates. A lid was used further to prevent the loss of the sample. Two types of experiments were carried out to the detect rheological properties of the dispersions under isothermal conditions: (1) a steady shear ranging from 0.01 to 500 s^−1^ and (2) a frequency sweep in the range of 0.1–100 rad/s at a constant strain of 5.0%.

### 2.4. Diffusing Wave Spectroscopy Microrheology

Microrheological measurements based on diffusing wave spectroscopy (DWS) were performed using a laboratory-made setup at 25 °C. The coherent source was a Spectra-Physics Cyan CDRH laser, operated at λ = 532 nm and an output power of 100 mW. The laser beam was expanded to approximately 1 cm at the sample. The scattered light was collected by an optical fiber and placed in the transmission geometry connected to two photomultiplier tubes (Hamamatsu, Japan) to measure a cross-correlation function of the scattered light. This method is used to circumvent the dead time of detector electronics and to reduce the after-pulsing effects, achieving correlation measurements with a very short delay time [28]. For DWS measurements, the polystyrene microspheres (500 nm in diameter) with 0.1 wt% content and without surface modification were dispersed in the dispersions as probe particles. The detailed procedure and theory are stated in Section S1 of the Supplementary Materials in Equations (S1)–(S6).

### 2.5. Small-Angle X-ray Scattering (SAXS)

The S-PFSA dispersions were transferred into custom-made SAXS cells (1 mm thick) that were then tightly sealed with glass windows (30 μm thick). The sample chamber was kept under vacuum (<0.005 Pa) at room temperature (about 20 °C) to eliminate ambient scattering. The profiles of the dispersions were then obtained using a SAXS point 2.0 instrument equipped with an EIGER R 1M detector (Anton Paar, Sydney, Austria), in which each sample was irradiated with an X-ray beam having a wavelength of 0.154 nm for 2 h at an SDD of 0.80 m. All the SAXS profiles were corrected for exposure time, transmittance, sample thickness, cell background, and solvent scattering and also normalized to the absolute intensity with the custom-made data reduction package Red2D on a scientific data analysis software (Igor Pro 8, Wave Metrics, Lake Oswego, OR, USA).

## 3. Results and Discussion

### 3.1. Microstructure of S-PFSA

**Figure 1:**
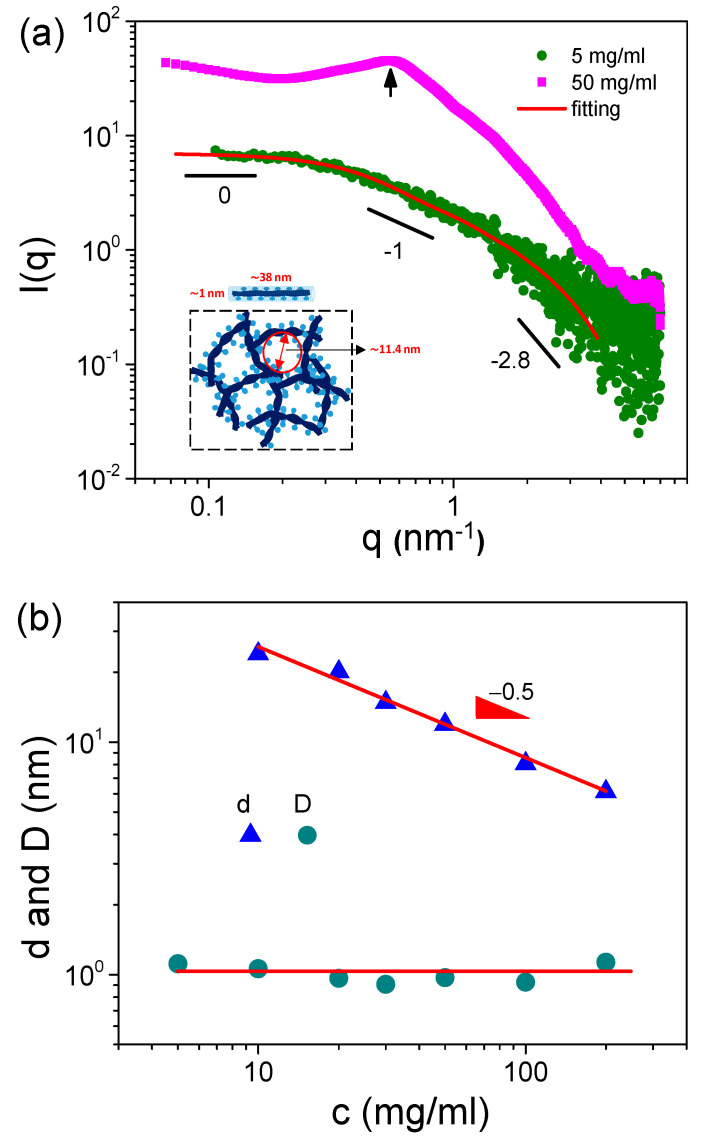
(**a**) SAXS profiles of S-PFSA dispersions at various polymer concentrations and the corresponding fitting curves based on cylindrical model. (**b**) Effect of S-PFSA concentration on colloidal particle distance and lateral size.

It is well known that S-PFSA cannot be fully dissolved in any solvent, but it can be dispersed as colloidal particles [29,30]. Therefore, it is necessary to obtain structural information regarding S-PFSA dispersions. Figure 1a shows the SAXS profiles of S-PFSA dispersions in water/2-propanol (50/50 vol/vol). At 5 mg/mL, the scattering intensity shows I(q)~q^−1^ at the intermediate q range, as expected for polyelectrolytes in salt-free water. The q dependence of the scattering intensity shifts from I(q)~q^−1^ to I(q)~q^−2.8^ with increasing q, suggesting that the S-PFSA particles have a cylindrical morphology [31,32]. In addition, the q^−2.8^ scaling also indicates an indistinct interface between the S-PFSA particles and the solvent, which is characteristic of amphiphilic colloidal aggregates. The scattering profiles suggest that the S-PFSA powders are not fully dissolved in water/2-propanol but rather are dispersed as cylindrical colloidal particles. Within these colloidal particles, the perfluorinated backbones of the S-PFSA interact with one another, while the sulfonate groups are in contact with the solvent molecules. This type of aggregated structure is very common in polysaccharides such as cellulose derivatives, which consist of a core of laterally aggregated backbone and dangling side chains [33].

The length (L) and lateral size (diameter, D) of the S-PFSA colloidal particles can be estimated based on the cylindrical model [34],

(1) $$P(q)=\frac{scale}{\pi r^{2}L}(\frac{z+1}{z+2})\int_{0}^{\infty }f(r)dr\int_{0}^{\pi /2}F^{2}(q,\alpha )\sin\alpha d\alpha$$

(2) $$F(q,\alpha )=2V_{cyl}\Delta \rho \frac{\sin((qL\cos\alpha )/2)}{(qL\cos\alpha )/2}\frac{J_{1}(qr\sin\alpha )}{qr\sin\alpha }$$

where the term “scale” is a scaling factor relative to intensity units of cm^−1^, *L* is the cylindrical length, *f*(*r*) is the normalized Schulz distribution of radius *r* (*p* = *σ*_r_/$\bar{r}$, where *p* is the polydispersity, *σ*_r_ is the root-mean-square deviation from the mean radius $\bar{r}$), *z* is a parameter related to the width of distribution and the relative polydispersity of radius as *z* = 1/(*σ*_r_/*r*)^2^ − 1, *V_cyl_* is the cylindrical volume, Δ*ρ* is the scattering length density difference, *α* is the angle between the cylindrical axis and the scattering vector *q*, and *J*_1_ is the first-order Bessel function. Figure 1a shows that the SAXS profile of the 5 mg/mL S-PFSA dispersion can be well fitted through the cylindrical model at a fixed cylindrical length of 38 nm and a radius of 0.5 nm. The resulting D value is approximately 1.0 nm, less than the lateral size of the L-PFSA, such as ~2.6 nm in water/ethanol (50/50 vol/vol) [35] and ~3.0 nm in water/2-propanol (80/20 vol/vol) [36]. The lateral diameter of a single S-PFSA chain was never measured. Here, we take the diameter of the sodium carboxymethyl cellulose chain, 0.3–0.4 nm [37], as the lateral size of a single S-PFSA chain. It can be estimated that the colloidal particle is composed of three to four S-PFSA chains.

Besides the colloidal particle length and diameter, the particle distance (d) was also obtained for S-PFSA dispersions at high polymer concentrations, such as 50 mg/mL, because of one interference peak in the scattering profile (Figure 1a). The d was subsequently calculated using Bragg’s law d = 2π/q, such as d = 8 nm for 100 mg/mL S-PFSA dispersion. It is noted that the d obtained from SAXS was smaller than the mesh size (ς) calculated from the rheological plateau modulus (G_e_), suggesting that there may be ion clusters formed among the meshes. In addition, the d decreases with increasing S-PFSA concentration (Figure 1b), suggesting that dense S-PFSA colloidal particles are formed at high polymer concentrations. A clear scaling relationship, d~c^−0.5^, was observed in the concentration range from 10 mg/mL to 200 mg/mL.

The SAXS data were further analyzed by multiscale structure fitting, in which the observed scattering profile is considered as a summation of various components [38,39,40,41,42],

(3) $$I(q)=\sum_{i=1}^{n}G_{i}\exp(-\frac{q^{2}{R_{gi}}^{2}}{3})+B_{i}\exp(-\frac{q^{2}{R_{g(i+1)}}^{2}}{3}){\left[\frac{(erf{\left(qR_{gi}/\sqrt{6}\right)}^{3}}{q}\right]}^{P_{i}}$$

where *G* is the classic Guinier prefactor, and *B* is a prefactor specific to the type of power law scattering, specified by the regime in which the exponent P falls. *R_gi_* and *R*_*g*(*i*+1)_ describe the average sizes of small-scale and large-scale structures. Generally, P < 3 is related to mass fractals, while 3 < P < 4 is related to surface fractals, and P > 4 is for diffuse interfaces. The *erf* is the error function.

**Figure 2:**
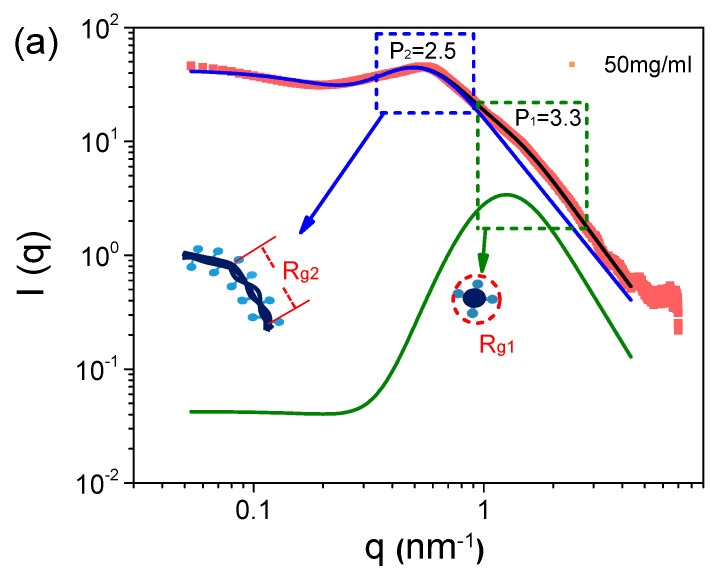

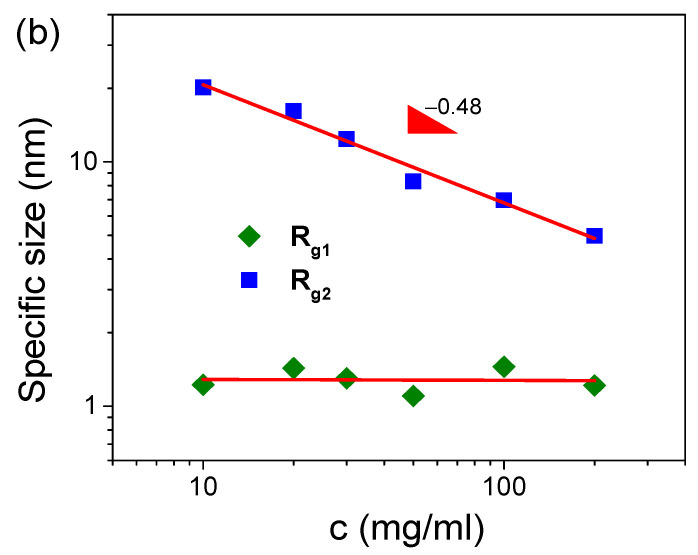
(**a**) The SAXS profile of 50 mg/mL S-PFSA dispersion and the corresponding multiscale structure fitting. (**b**) Effect of S-PFSA content on R_g1_ and R_g2_.

The scattering profile of the 50 mg/mL S-PFSA dispersion is well fitted using Equation (3), as shown in Figure 2a. The specific sizes, *R*_*g*1_ and *R*_*g*2_, are identified as the lateral size and correlation length (ξ) of the colloidal particles. In the low-q region, the lateral size of the colloidal particles was calculated to be 1.3 nm, closing to ~1 nm obtained through the cylindrical model. The correlation length was about 8.3 nm, much less than the length of the expanded colloidal particle, suggesting the collapse (semiflexible feature) of the S-PFSA colloidal particles under high polymer concentrations, similar to that observed for charged polysaccharides [43,44]. Furthermore, *R*_*g*1_ almost kept consistent with the increasing S-PFSA concentration and ξ decreased with the S-PFSA concentration, scaling as ξ~c^−0.48^ close to ~c^−0.5^. Note that the particle distance and the correlation length obey the same scaling law, suggesting that the increasing S-PFSA concentration has similar influence on the conformation and density of the S-PFSA colloidal particles. The same scaling law was also reported in polyelectrolyte solutions and confirmed by several theoretical studies [7,45,46,47].

Based on the SAXS studies, the microstructure of S-PFSA in water/2-propanol is proposed. Due to the amphiphilic feature of S-PFSA, semiflexible colloidal particles are formed with a hydrophobic associated core composed of perfluorinated backbones and a hydrophilic surface containing sulfonate groups that are in contact with the solvent molecules. At low polymer concentrations, the colloidal particles show an extended conformation like cylindrical shape due to electrostatic interaction, and the length and lateral diameter are about 38 nm and 1.0–1.3 nm. With an increase in the S-PFSA concentration, the colloidal particles collapse and the distance between the particles decreases, suggesting that much denser particles formed in the S-PFSA dispersions. Next, we explored the rheological properties of the S-PFSA dispersions to understand the effect of multiple interactions on the viscoelasticity of the S-PFSA dispersions.

### 3.2. Rheological Properties

**Figure 3:**
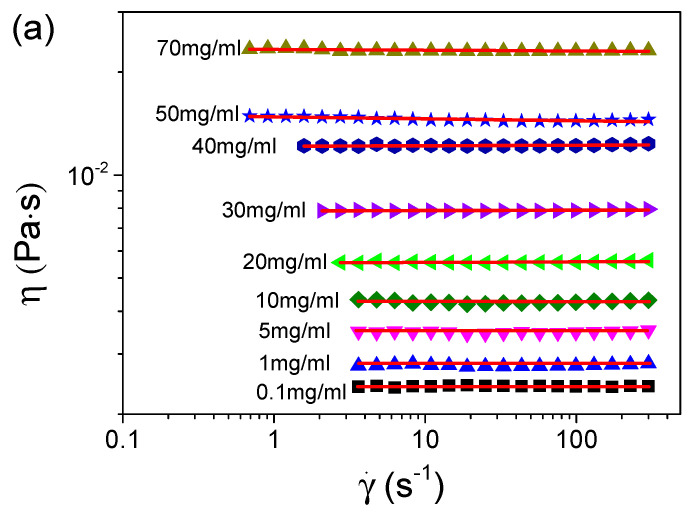

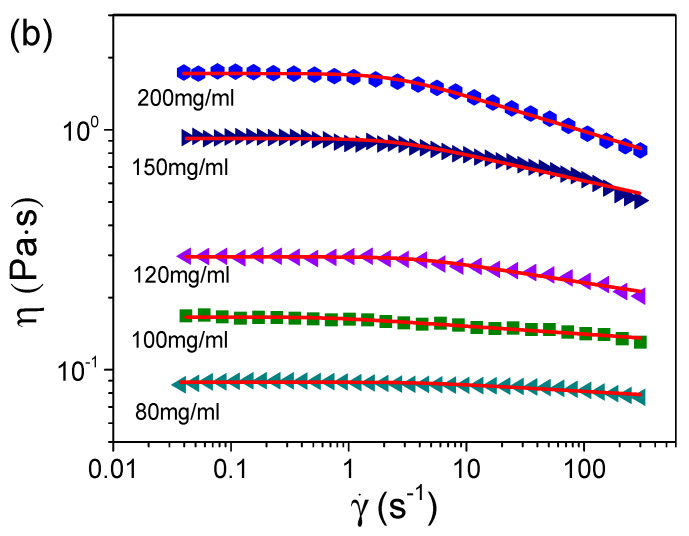
Dependence of viscosity on shear rate for S-PFSA dispersions with concentrations of (**a**) 0.1–70 mg/mL and (**b**) 80–200 mg/mL. These viscosity data were acquired by macrorheological measurements based on steady shear.

We first measured the viscosities of the S-PFSA dispersions through the steady shear approach. Figure 3 summarizes the viscosities of various S-PFSA dispersions as functions of the shear rate. The S-PFSA dispersions ranging from 0.1 to 70 mg/mL behaved as Newtonian fluids. In contrast, non-Newtonian behavior (also termed shear thinning) was observed as the S-PFSA content was increased to 80 mg/mL. The shear thinning became more pronounced with a further increase in the S-PFSA concentrations (c ≥ 100 mg/mL). The viscosity data can be fitted based on the Carreau model [48,49],

(4) $$\eta (\dot{\gamma })=\frac{\eta_{0}}{{\left(1+{\left(\tau \dot{\gamma }\right)}^{2}\right)}^{n/2}}$$

where *η*_0_ is the zero-shear viscosity, $\dot{\gamma }$ is the shear rate, *τ* is the relaxation time, and *n* is the power law exponent of apparent viscosity in the shear thinning regime. The dependence of viscosity on the shear rate in S-PFSA dispersions suggests the formation of weak microstructures, which are possibly composed of transient physical interactions (such as hydrophobic interaction) or entanglement between the S-PFSA colloidal particles. When a certain amount of shear is applied, these weak interactions are broken, causing a temporal decrease in the viscosity.

**Figure 4:**
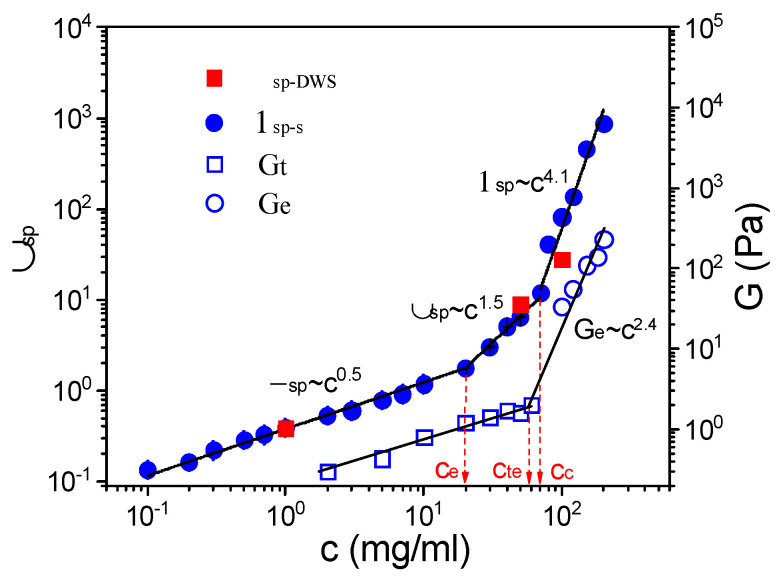
Dependence of η_sp_, G_e_, and G_t_ on S-PFSA concentration, where η_sp-s_ (filled circle) and η_sp-DWS_ (filled square) are obtained from macrorheology and DWS microrheology, respectively. The solid lines represent guides to the scaling relationships in semidilute unentangled regime (η_sp_~c^0.5^), semidilute entangled regime (η_sp_~c^1.5^), and concentrated regime (η_sp_~c^4.1^).

The specific viscosity (η_sp_), defined in terms of the expression η_sp_ = (η_0_ − η_s_)/η_s_, where η_s_ is the viscosity of solvent, is one of the most important parameters characterizing a polymer solution. For samples that do not exhibit shear thinning, an average over all the viscosity values is taken as η_0_. Because of shear thinning under high polymer concentrations, η_0_ is influenced by the shear rate. Thus, η_0_ is obtained through the Carreau model. The dependence of η_0_ on the polymer concentration for the S-PFSA dispersions in water/2-propanol exhibited a piecewise power law relationship (Figure S4). The η_s_ obtained by steady shear is shown in Figure S5. The values of η_sp_ obtained from steady shear (η_sp-s_) are plotted as a function of the S-PFSA content (*c*) in Figure 4. Three distinct regimes with different scaling relationships, η_sp_~c^0.5^ for c ≤ 20 mg/mL, η_sp_~c^1.5^ for 20 < c ≤ 70 mg/mL, and η_sp_~c^4.1^ for 70 mg/mL < c ≤ 200 mg/mL, can be identified. The specific viscosity was also acquired by performing diffusing wave spectroscopy (DWS) microrheological measurements. It was found that the η_sp_ obtained from DWS (η_sp-DWS_) remained consistent with the steady shear and obeyed the same scaling relationships, suggesting that the Carreau model provides a reliable estimation.

The scaling theory proposed by Dobrynin et al. from the Rouse model derives the dependence of the specific viscosity on the polymer concentration scales as c^0.5^ for salt-free semidilute unentangled polyelectrolyte solutions, which is the well-known Fuoss law. As the polymer content exceeds the entanglement concentration, the polymer chains strongly overlap. The motion of the chains becomes topologically constrained by the presence of neighboring chains, known as entanglement effects, in which the chains are not able to pass through each other. The classical polyelectrolyte solution theory predicts a power law exponent of 1.5. The scaling relationship between the specific viscosity and the S-PFSA concentration conforms to the theory of polyelectrolyte solutions. The corresponding regimes are denoted as the semidilute unentangled regime and the semidilute entangled regime, and the crossover of η_sp_~c^0.5^ and η_sp_~c^1.5^ is defined as the entanglement concentration, c_e_. With a further increase in the polymer content, the specific viscosity has a strong upturn accompanied by a high power law exponent of 4.1, which is close to the theory’s prediction (4.2) for a neutral concentrated polymer solution in a good solvent [50,51]. Thus, the regime of η_sp_~c^4.1^ was identified as the concentrated regime, and the onset concentration of the concentrated S-PFSA dispersion was marked as c_c_.

However, many experimental results for polyelectrolyte solutions show a deviation of c_e_ from the theory predictions. For example, for polystyrene sulfonate solutions, c_e_ obtained from the viscosity data follows a power law of N^−0.77^ (N is the polymerization degree) [52,53], inconsistent with the scaling theory prediction for salt-free polyelectrolytes (c_e_~N^−2^) [54], while the exponent of concentration dependence of viscosity matches perfectly with scaling theory. These results indicate that the widely used crossover method to obtain c_e_ by detecting the dependence of the specific viscosity on the concentration, where the transition of η_sp_~c^0.5^ to η_sp_~c^1.5^ occurs, might underestimate or overestimate c_e_.

**Figure 5:**
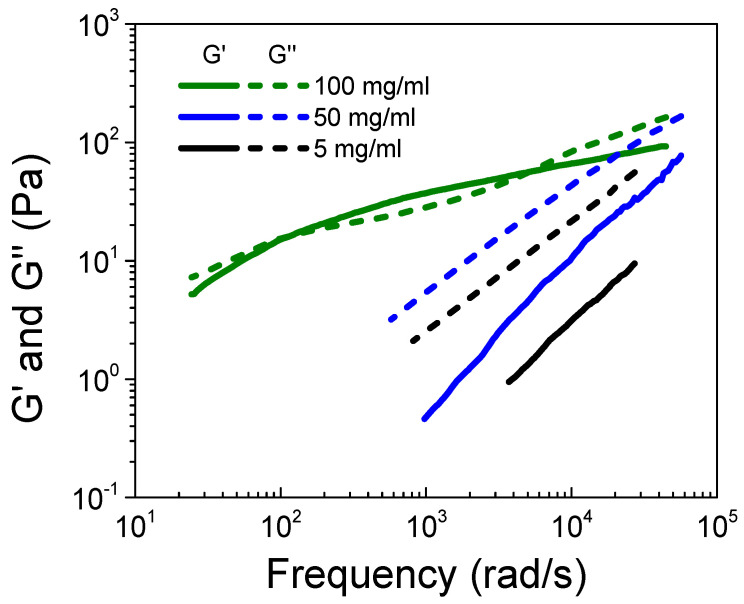
Storage modulus (G’) and loss modulus (G”) obtained from DWS microrheological measurements for the S-PFSA dispersions at 5, 50, and 100 mg/mL.

The easiest way to study entanglement property is through linear viscoelasticity (LVE) analysis. However, with commercially available rheometers, the LVE response of a polyelectrolyte solution is limited to the terminal regime. The crossover of storage and loss moduli at low frequency usually fails to detect the entanglement of polymer chains. Herein, we adopted the diffusing wave spectroscopy (DWS) microrheological technique, which can provide dynamic information at higher frequency up to 10^5^ rad/s. Figure 5 displays the storage modulus (G’) and the loss modulus (G”) obtained from the MSD of the probe particles based on the GSER equation (Equation (S3)) for the S-PFSA dispersions at c = 5, 50, and 100 mg/mL, which locate in the semidilute unentangled, semidilute entangled, and concentrated regimes, respectively. It is apparent that an increase in the S-PFSA content induces a transition from viscosity to viscoelasticity. Specifically, the dispersions show a typical liquid behavior at c = 5 and 50 mg/mL, where G” is larger than G’ within the probed frequency range. As the S-PFSA concentration is increased to 100 mg/mL, two crossover points between G’ and G” are observed. G’ exceeds G” at the intermediate frequency, corresponding to the plateau zone, which highlights the emergence of elasticity of the dispersion.

The LVE analysis indicates that the S-PFSA dispersion of 50 mg/mL locating in the semidilute entangled regime is not truly entangled as G” is greater than G’. The increase in viscosity may be attributed to other mechanisms. In the semidilute unentangled regime, the electrostatic interactions expand the chain conformation, leading to an increase in the solution viscosity. The increasing S-PFSA content results in a decrease in the particle distances. The weak hydrophobic interaction between the associated backbones becomes distinct, which contributes to an increase in the dispersion viscosity. At higher polymer concentrations, the electrostatic interactions are screened, and the role of entanglement is manifested. Similar results were observed in the aqueous solution of sodium carboxymethyl cellulose (NaCMC) with various hydrophobic substitution degrees [55]. Therefore, we think that the viscosity data underestimate the entanglement concentration of S-PFSA dispersions. The increase in viscosity at c_e_ might be induced by the hydrophobic interactions between the associated backbones, which produce a strong dependence of the power law exponent in the semidilute unentangled regime. The obtained c_e_ is the result of an adopted definition of the entanglement threshold, at which the concentration dependence of η_sp_ switches from c^0.5^ to c^1.5^. This change in the viscosity dependence of the S-PFSA dispersions does not correlate with the appearance of entanglements.

**Figure 6:**
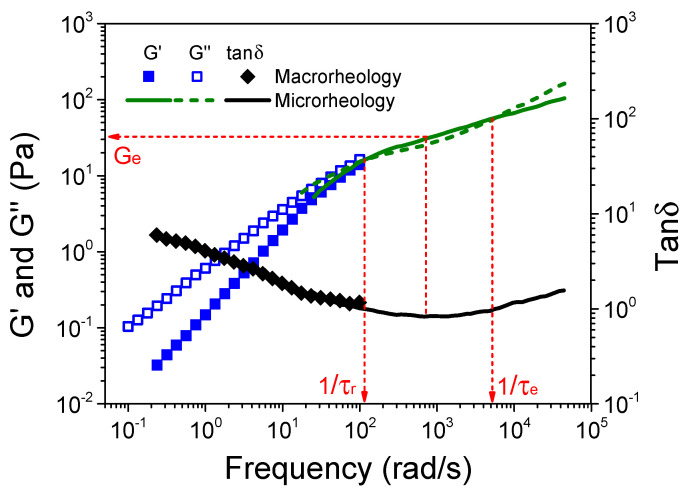
Frequency dependence of G’, G”, and tanδ for the S-PFSA dispersion of 100 mg/mL. The dot data were obtained from macrorheology, and the line data were measured by DWS microrheology. The red dashed arrows guide the determination of G_e_, τ_r_, and τ_e_.

Figure 6 shows the variation of G’, G”, and the loss tangent (tanδ) as a function of frequency for the S-PFSA dispersion of 100 mg/mL by combining macrorheology with DWS microrheology. The rheological data obtained from two rheological technologies maintain good consistency at the overlapping region. Most importantly, based on two complementary techniques, the experimental results cover a wide frequency range from 10^−1^ to 10^5^ rad/s, where the terminal flow behavior at low frequency and the rubbery plateau regime between the two crossovers of G’ and G” are better demonstrated. With the full rubbery plateau regime of dynamic moduli, we estimate the terminal relaxation time τ_r_ (5.2 × 10^−2^ s) and the entanglement strand relaxation time τ_e_ (1.2 × 10^−3^ s) as the inverse of the crossover frequency of G’ and G” at low and high frequencies, respectively. The plateau modulus G_e_ = 34.3 Pa is estimated from the storage modulus at a frequency at which tanδ displays a minimum (the so-called MIN method) [56].

**Figure 7:**
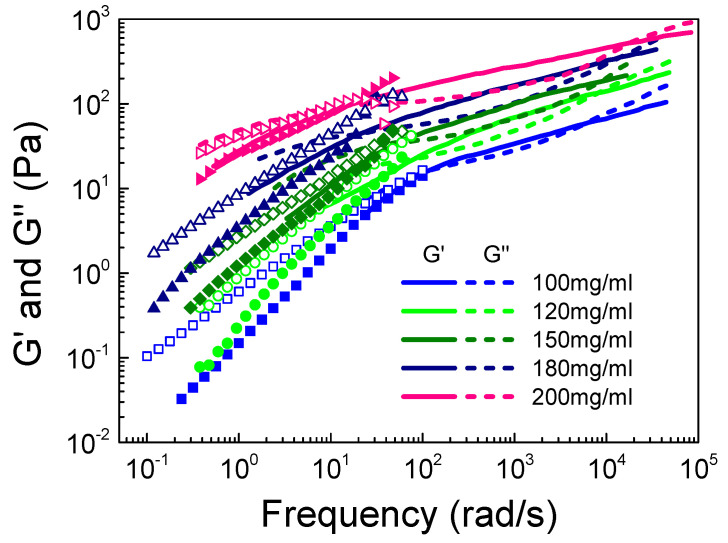
Frequency dependence of G’ and G” on S-PFSA concentration in the concentrated regime. The dot data were obtained from macrorheology, and the line data were measured by DWS microrheology.

We first explored the effect of the S-PFSA content on the entanglement dynamic, since τ_r_ and τ_e_ directly relate to the relaxation process of semiflexible S-PFSA colloidal particles. Figure 7 shows the viscoelastic spectra of dispersions with different S-PFSA concentrations (100 mg/mL ≤ c ≤ 200 mg/mL) measured by macrorheology and DWS microrheology. It is noted that G’ and G” were obtained by converting the MSD curves (Figure S6), which show a twist at the concentration of 100 mg/mL, suggesting the presence of elasticity. It is reasonable that the values of G’ and G” increase with increasing S-PFSA concentration, since the number of colloidal particles increase with increasing c. The crossovers of G’ and G” at low- and high-frequency regions, respectively, move to the lower and higher values, suggesting that the τ_r_ of the particles increases while the τ_e_ of the entanglement strand decreases with increasing S-PFSA concentration. The combined microrheological measurements also capture the expected increase in the width of the rubbery plateau with increasing S-PFSA.

To visualize the behavior of the viscoelastic parameters, G_e_ as a function of the S-PFSA concentration in the concentrated regime is exhibited in Figure 4. First of all, the mesh size (ς) can be obtained based on G_e_ = k_B_T/ς^3^, where k_B_ and T denote Boltzmann’s constant and absolute temperature, respectively. It was found that ς decreased from ~49 nm to ~24 nm when the S-PFSA concentration increased from 100 mg/mL to 200 mg/mL. In addition, the relationship of G_e_ with the S-PFSA concentration showed a power law dependence, G_e_~c^2.4^. Graessley and Edwards studied the viscoelastic response of entangled solutions of neutral linear polymers, such as polystyrene and polybutadiene, and found that G_e_ increases with polymer concentration as a power law exponent of 2.3 [57]. However, Matsumoto et al. found the scaling relation G_e_~c^1.5^ for the semidilute entangled poly(sodium styrenesulfonate) solution [58]. The dependence of G_e_ on S-PFSA agreed with that in neutral polymer solutions, which is consistent with the scaling relation of specific viscosity with S-PFSA concentration (ɳ_sp_~c^4.1^), indicating that the S-PFSA dispersions under the concentrated regime show the characteristics of a neutral polymer solution. In addition, extrapolating G_e_~c^2.4^ back to the terminal modulus (G_t_), which is determined from the Carreau model based on G_t_ = η_o_/τ, gives the crossover concentration (c_te_). The c_te_ represents the transition from viscosity to viscoelasticity of polymer solution and is considered as the onset of entanglement. It was found that the c_te_ (58 mg/mL) was higher than c_e_ (20 mg/mL), suggesting that c_e_ from the concentration dependence of η_sp_ changing from c^0.5^ to c^1.5^ is not the true entanglement concentration for the S-PFSA dispersions. The true entanglement of S-PFSA colloidal particles occurs at a higher polymer concentration.

**Figure 8:**
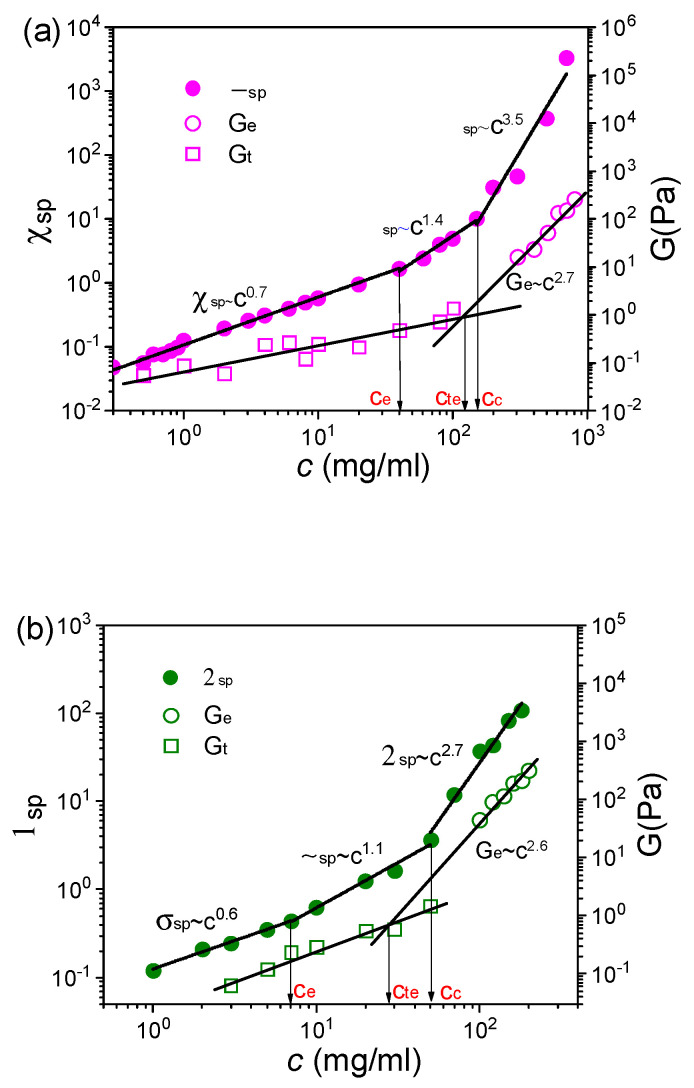
The relationships of η_sp_, G_e_, and G_t_ with *c* for (**a**) the L-PFSA dispersions in water/2-propanol and (**b**) the S-PFSA dispersions in water/ethanol.

To further confirm the above experimental results, the rheological responses of long-side-chain perfluorosulfonic acid (L-PFSA, EW = 960) dispersions in water/2-propanol and S-PFSA dispersions in water/ethanol were studied based on macrorheology and DWS microrheology. The flow curves of L-PFSA in water/2-propanol and S-PFSA in water/ethanol with different polymer concentrations are shown in Figures S7 and S10, and the change in η_o_ with the polymer concentration shows a piecewise power law relationship (Figures S8 and S11). In addition, the dependence of η_sp_ on the polymer concentration can be divided into three regimes, and the corresponding scaling relationships, η_sp_~c^0.7^, η_sp_~c^1.4^ and η_sp_~c^3.5^ for the L-PFSA dispersion in water/2-propanol (Figure 8a) and η_sp_~c^0.6^, η_sp_~c^1.1^ and η_sp_~c^2.7^ for the S-PFSA dispersion in water/ethanol (Figure 8b), are close to the theory predictions. These regimes are denoted as the semidilute unentangled, semidilute entangled, and concentrated regimes, respectively. However, the LVE demonstrates that G” is larger than G’ for the 100 mg/mL L-PFSA dispersion in water/2-propanol (Figure S9) and the 20 mg/mL S-PFSA dispersion in water/ethanol (Figure S12), representing a typical liquid behavior. Extrapolating G_e_ back to G_t_, c_te_ was obtained. It is found from Figure 8a,b that the c_te_ was larger than the c_e_ for both L-PFSA dispersions in water/2-propanol and S-PFSA dispersions in water/ethanol. These results further indicated that the c_e_ obtained from the concentration dependence of η_sp_ changing from c^0.5^ to c^1.5^ was underestimated and that the true entanglement takes place at a high polymer concentration (approaching c_c_).

**Figure 9:**
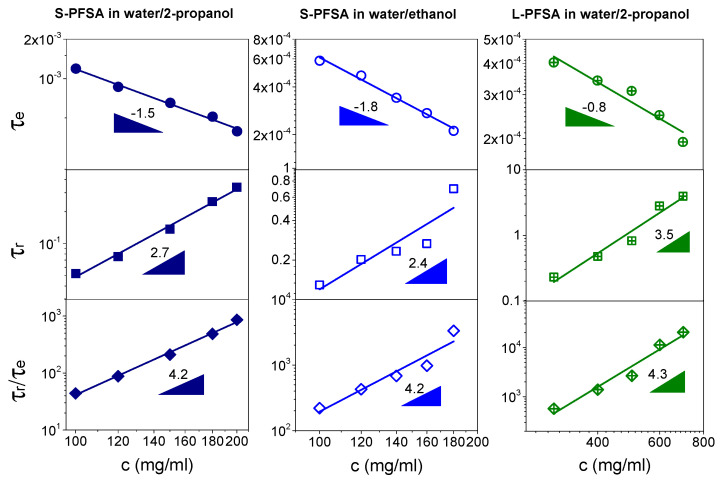
Dependence of τ_e_, τ_r_, and τ_r_/τ_e_ on polymer concentration in the concentrated regime for the S-PFSA dispersions in water/2-propanol and water/ethanol as well as the L-PFSA dispersion in water/2-propanol.

Figure 9 shows the effect of the polymer concentration on the τ_e_ and τ_r_ in the concentrated regime. In general, the τ_e_ decreases and the τ_r_ increases with increasing polymer concentration. For the S-PFSA dispersions in different solvents, the τ_e_ and τ_r_ showed a similar power law dependence, τ_e_~c^−1.5^ and τ_r_~c^2.7^ in water/2-propanol and τ_e_~c^−1.8^ and τ_r_~c^2.4^ in water/ethanol. However, the L-PFSA dispersion demonstrated the scaling relationships, τ_e_~c^−0.8^ and τ_r_~c^3.5^, indicating that the length of the side chain has an important effect on the relaxation of the colloidal particles. The possible reason is that the hydrophilicity of the side chain decreased with the increasing length of the side chain, since the side chain is mainly composed of carbon and fluorine atoms besides the sulfonic groups. The hydrophobic side chains promote the association of the polymer backbone, leading to a large lateral size. This inference was confirmed by SAXS, where the lateral size of S-PFSA was about 1–1.3 nm, which is lower than that (~2.6 nm) of L-PFSA (EW = 1100 g/mol) reported by Yamaguchi et al. [35]. Meanwhile, it can be found that the c_te_ (122 mg/mL) in the L-PFSA dispersion was higher than those (58 mg/mL in water/2-propanol and 27 mg/mL in water/ethanol) in the S-PFSA dispersions, indicating that much more of the L-PFSA is associated into the colloidal particles. In addition, the scaling exponents of τ_e_ and τ_r_ in both the S-PFSA and L-PFSA dispersions are different from the scaling prediction of the neutral polymer solution in the semidilute entangled regime, suggesting that the relaxation behavior of S-PFSA and L-PFSA in the concentrated regime is influenced by the hydrophobic interaction of their polymer backbones.

In a fixed-dispersion system, the effect of hydrophobic interaction on τ_r_ and τ_e_ is approximate, owing to the same chain structures of cylindrical colloidal particles and entanglement strands. Therefore, the ratio of τ_r_/τ_e_, proposed by Han and Colby [59], can be adopted to remove the effect of the hydrophobic interaction on the dynamics of the colloidal particles. Figure 9 further shows the concentration dependence of τ_r_/τ_e_ for the S-PFSA and L-PFSA dispersions. The value of τ_r_/τ_e_ magnifies with increasing polymer concentration but has a similar power law dependence, τ_r_/τ_e_~c^4.2^ for S-PFSA and τ_r_/τ_e_~c^4.3^ for L-PFSA, approaching that of neutral polymer solutions in the semidilute entangled regime (power law exponent 4.0) [57]. Therefore, the deviation of scaling laws for τ_r_ and τ_e_ in the concentrated regime is attributed to the hydrophobic interaction of the polymer backbones, which was not considered in the polyelectrolyte solution theory, e.g., in Dobrynin et al.’s model.

## 4. Conclusions

In this study, we systematically investigated the structure and rheological properties of S-PFSA, which is an important fluoric polymer for proton exchange membranes in fuel cells. In water/2-propanol, S-PFSA disperses as cylindrical colloidal particle with a length of ~38 nm and a lateral diameter of 1–1.3 nm. The relevant length (ξ) reduces as the content of S-PFSA increases and follows the relationship ξ~c^−0.5^, consistent with the prediction of scaling law. The electrostatic interactions primarily affect the conformation of S-PFSA and solution viscosity at a low concentration due to repulsion. The hydrophobic interactions produce a further increase in the dispersion viscosity, leading to strong exponent dependence of the specific viscosity on the concentration. Under a higher polymer concentration, the electrostatic interactions were screened, and the role of entanglement was manifested. By extrapolating the plateau modulus to the terminal modulus, a more accurate entanglement concentration was obtained. And it was greater than the entanglement concentration determined from the viscosity dependence, suggesting that the scaling theory, in which the transition of η_sp_~c^0.5^ to η_sp_~c^1.5^ occurs, underestimates the true entanglement concentration. The rheological properties of S-PFSA in water/ethanol and L-PFSA in water/2-propanol further confirm the influence of the hydrophobic interactions on the viscosity and viscoelasticity of the dispersions. These results suggest that the PFSA dispersions have a complex scaling law due to multiple interactions.

## Data Availability

Data will be made available on request.

## Supplementary Materials

The following supporting information can be downloaded at https://www.mdpi.com/article/10.3390/polym16131863/s1: Figure S1: NMR spectrum of S-PFSA and L-PFSA. Figure S2: FTIR spectrum of S-PFSA dispersions. Figure S3: Schematic of dispersion preparation. Figure S4: Zero-shear viscosity (η_0_) for S-PFSA dispersions in water/2-propanol. Figure S5: Viscosity for solvent water/2-propanol and water/ethanol. Figure S6: Mean square displacement master curves of S-PFSA dispersions in water/2-propanol. Figure S7: Viscosity for the L-PFSA dispersions in water/2-propanol. Figure S8: η_0_ for L-PFSA dispersions in water/2-propanol. Figure S9: G’ and G” for the L-PFSA dispersions in water/2-propanol. Figure S10: Viscosity for S-PFSA dispersions in water/ethanol. Figure S11: η_0_ for S-PFSA dispersions in water/ethanol. Figure S12: G’ and G” for S-PFSA dispersion in water/ethanol. Table S1: Parameters for cylindrical model fitting. Table S2: Parameters for multiscale structure fitting. References [60,61,62,63,64,65,66] are cited in the Supplementary Materials.
