# Supplementary material for: Microstructures and Rheological Properties of Short-Side-Chain Perfluorosulfonic Acid in Water/2-Propanol
Source: Polymers (Basel). 2024 Jun 29;16(13):1863. doi: 10.3390/polym16131863 (PMC11244402; doi:10.3390/polym16131863)
Supplement: Supplementary file 1 [file polymers-16-01863-s001.zip › polymers-3079065-supplementary.pdf]

Supporting information

Microstructures and Rheological Properties of Short-Side-Chain Perfluorosulfonic Acid in Water/2-Propanol

Yan Qiu [1], Xinyang Zhao [1], Hong Li [2], Sijun Liu [1,*] and Wei Yu [1,*]

[1] Advanced Rheology Institute, Department of Polymer Science and Engineering, Shanghai Jiao Tong University, Shanghai 200240, China
[2] Shanghai Electrochemical Energy Devices Research Center, School of Chemistry and Chemical Engineering, Shanghai Jiao Tong University, Shanghai 200240, China
* Correspondence: liusijun@sjtu.edu.cn (S.L.); wyu@sjtu.edu.cn (W.Y.)

S1. Diffusing wave spectroscopy microrheology

S-PFSA and L-PFSA dispersions for DWS measurements were loaded into cuvettes for fluorescence spectroscopy (L=4 mm in path length). The obtained intensity autocorrelation function $g^{(2)}(t)$ was converted into the field autocorrelation function $g^{(1)}(t)$ by Siegert relation, $g^{(2)}(t)=1+[g^{(1)}(t)]^2$, then the mean-square displacement (MSD) of the probe particles, $\langle\Delta R^2(t)\rangle$, was calculated by numerically solving the following equations [1-2]

$$g^{(1)}(t)=\frac{\frac{L}{l^*}+\frac{4}{3}}{\frac{z_o}{l^*}+\frac{2}{3}}\times\frac{\sinh\left[\frac{z_o}{l^*}r(t)\right]+\frac{2}{3}r(t)\cosh\left[\frac{z_o}{l^*}r(t)\right]}{(1+\frac{4}{9}r(t)^2)\sinh\left[\frac{L}{l^*}r(t)\right]+\frac{2}{3}r(t)\cosh\left[\frac{L}{l^*}r(t)\right]} \tag{S1}$$

where $r(t)=\sqrt{(2\pi/\lambda)^2\left\langle\Delta R^2(t)\right\rangle}$ is the root of MSD nondimensionalized by wavelength, l^* is the sample transport mean free path of the scattered light determined from the values of transmission intensity of the sample and a reference sample (water) whose l^* is known. For this system, l^* was found to be 1690 μm for 0.1 % of probes. z_o is the distance that the light

must travel through the sample before becoming randomized, here it is set $z_o = l^*$. The measured multiply scattered light signal was found to be ergodic for all the samples studied.

By using the mean square displacement of a probe particle of radius R_p, the frequency dependence of the complex shear modulus can be obtained from the generalized Stokes−Einstein equation [3-4]

$$G(s) = \frac{k_B T}{\pi R_p s \left\langle \Delta r^2(s) \right\rangle} \tag{S2}$$

where G(s) is the viscoelastic spectrum as a function of Laplace frequency s, k_B is the Boltzmann constant, T is temperature, and $\left\langle \Delta r^2(s) \right\rangle$ is the Laplace transform of the mean square displacement $\left\langle \Delta r^2(t) \right\rangle$. This equation can be expressed in the Fourier frequency domain [5]

$$G^*(\omega) = \frac{k_B T}{\pi R_p i\omega \overline{\zeta} \left\langle r^2(t) \right\rangle} \tag{S3}$$

where $\overline{\zeta} \left\langle r^2(t) \right\rangle$ is Fourier transform of mean square displacement. Assuming a local power law form for the mean square displacement, we can express $G^*(\omega)$ as a function of the experimentally measured mean square displacement $\left\langle \Delta r^2(t) \right\rangle$

$$G^*(\omega) = \frac{k_B T}{\pi R_p \left\langle \Delta r^2(\frac{1}{\omega}) \right\rangle \Gamma(1 + a(\omega))} \tag{S4}$$

where $t = 1/\omega$, $\alpha(t) = \partial[\ln<\Delta r^2(t)>]/\partial[\ln t]$ is the slope of the graph of $\left\langle \Delta r^2(t) \right\rangle$ plotted against time to logarithmic scales evaluated at t. Γ is the gamma function which serves as the conversion factor of the transform. Finally, we obtain

$$G'(\omega) = G^*(\omega)\cos\left[\pi \cdot \alpha(\omega)/2\right] \quad \text{(S5)}$$

$$G''(\omega) = G^*(\omega)\sin\left[\pi \cdot \alpha(\omega)/2\right] \quad \text{(S6)}$$

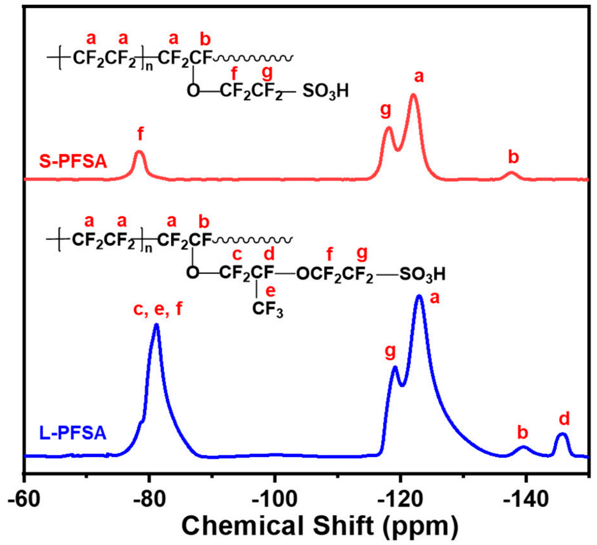

Figure S1. Nuclear magnetic resonance (^{19}F NMR) spectrum of S-PFSA and L-PFSA (DMSO as solvent). The peaks and their corresponding structures are marked with the same letters.

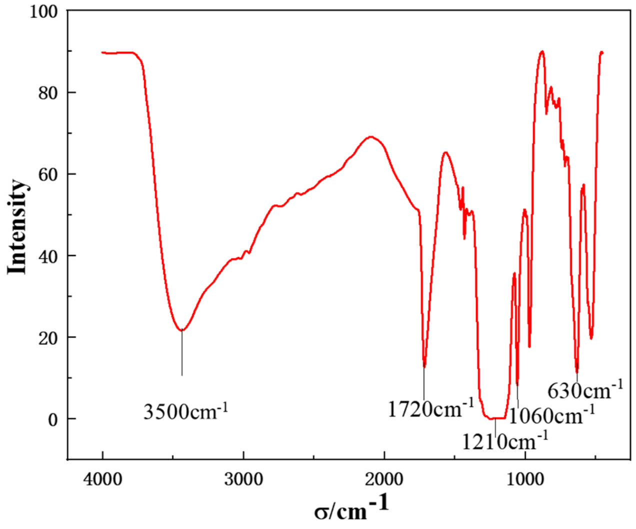

Figure S2. FTIR spectrum of S-PFSA dispersions. The characteristic peaks at 1210 cm^{-1} and 1060 cm^{-1} are attributed to the stretching vibration of -CF_2-CF_2- and the symmetric stretching vibration of -S=O. The peak at 1720 cm^{-1} is related to -OH bond on the sulfonic acid group. The peak at 630 cm^{-1} is assigned to the bending vibration of -CF_2- [6-7].

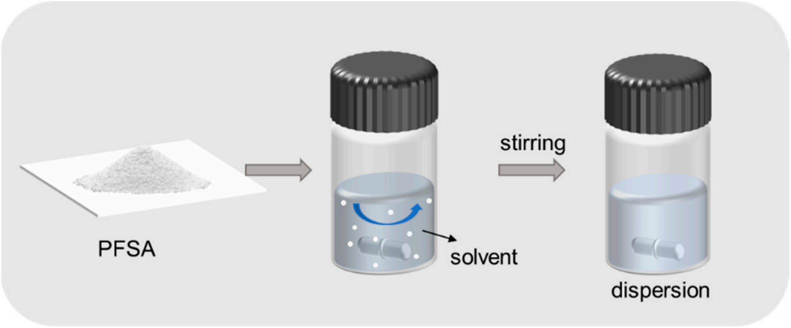

Figure S3. Schematic illustration for the preparation of PFSA dispersions.

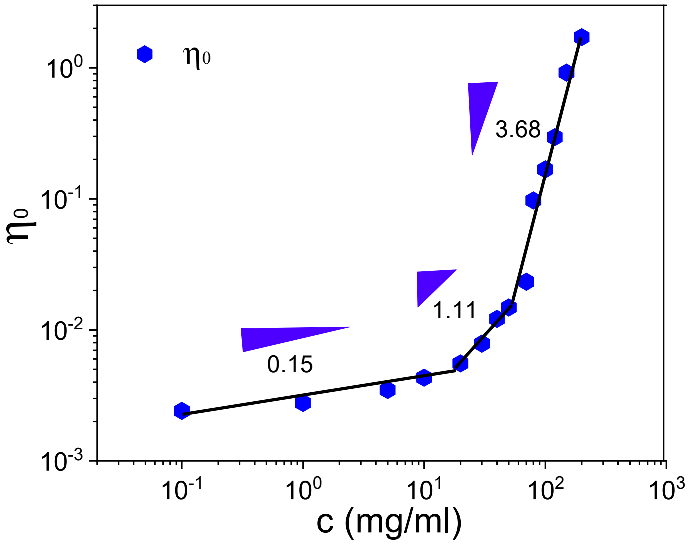

Figure S4. Dependence of zero-shear viscosity (η_o) on concentration for the S-PFSA dispersions in water/2-propanol with concentration ranging from 0.1 mg/ml to 200 mg/ml.

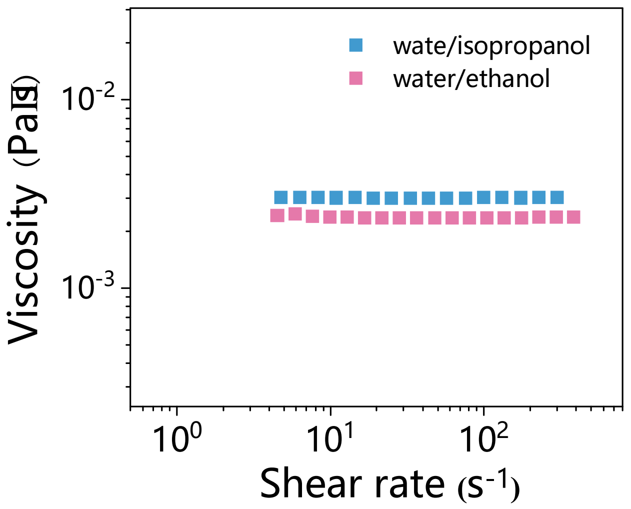

Figure S5. Dependence of viscosity on shear rate for the water/2-propanol and water/ethanol mixture solvents.

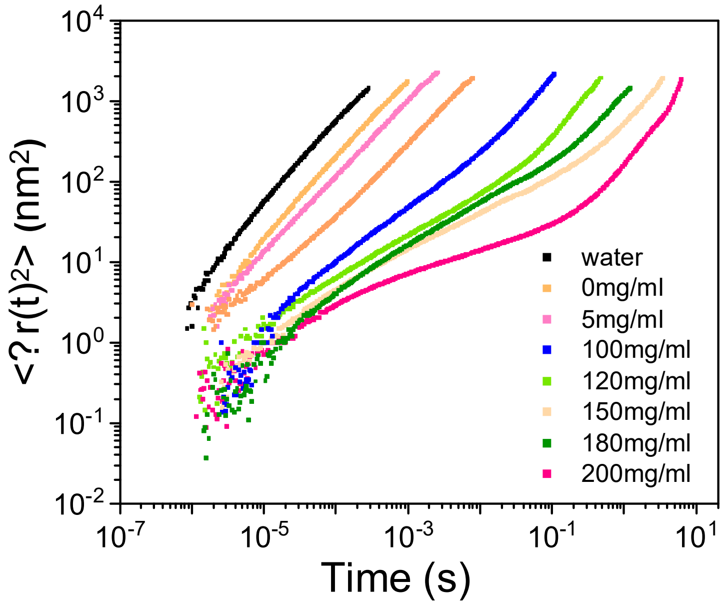

Figure S6. Mean square displacement master curves of the S-PFSA dispersions in water/2-propanol with concentrations of 0-200 mg/ml.

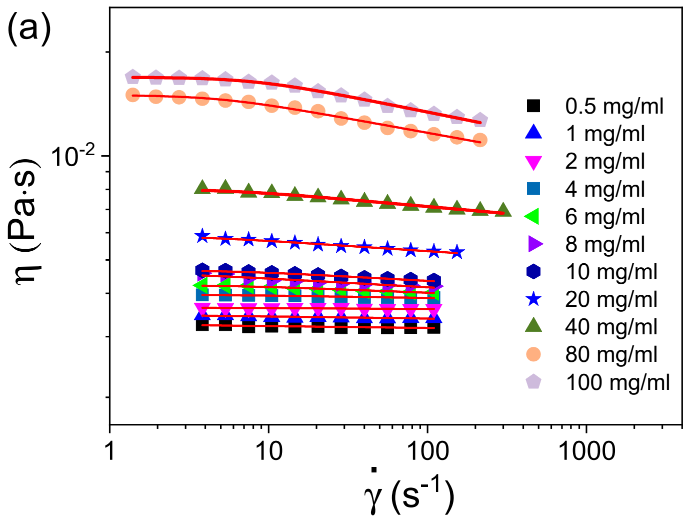

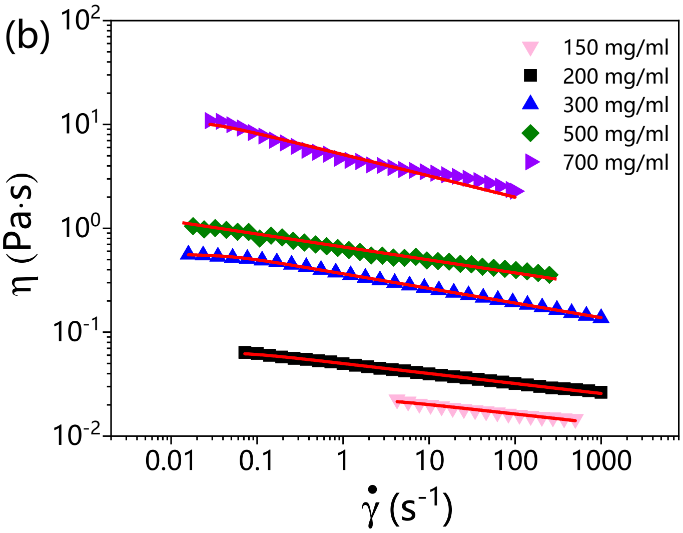

Figure S7. Dependence of viscosity on shear rate for the L-PFSA dispersions in water/2-propanol with concentrations of (a) 0.5-100 mg/ml and (b) 150-700 mg/ml. These viscosity data were acquired by macrorheological measurements based on steady shear.

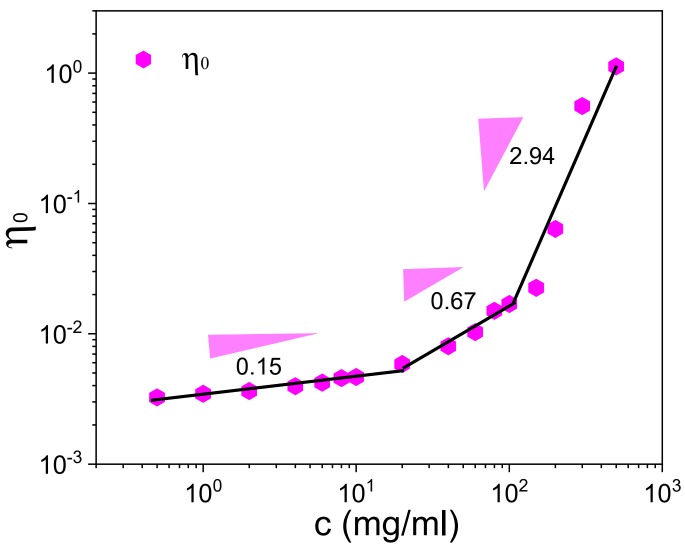

Figure S8. Dependence of zero-shear viscosity (η_o) on concentration for the L-PFSA dispersions in water/2-propanol with concentrations of 0.5-700 mg/ml.

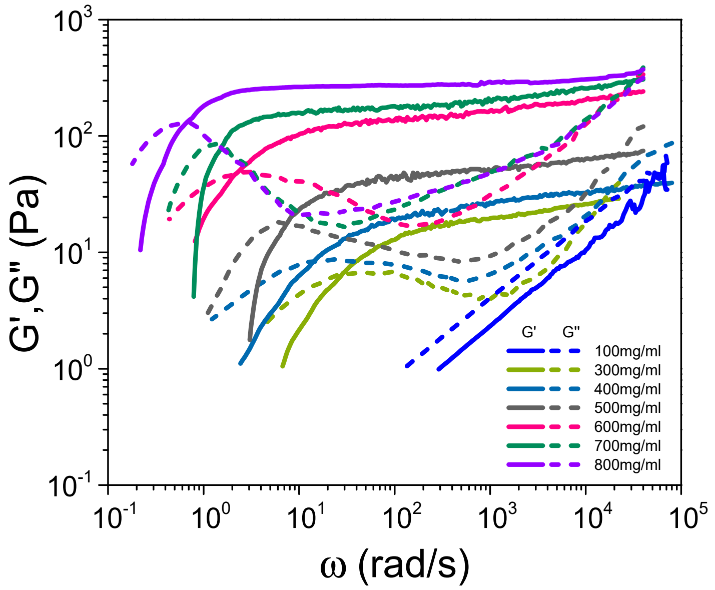

Figure S9. Frequency dependence of G′ and G″ for the L-PFSA dispersions in water/2-propanol obtained from DWS microrheological measurements.

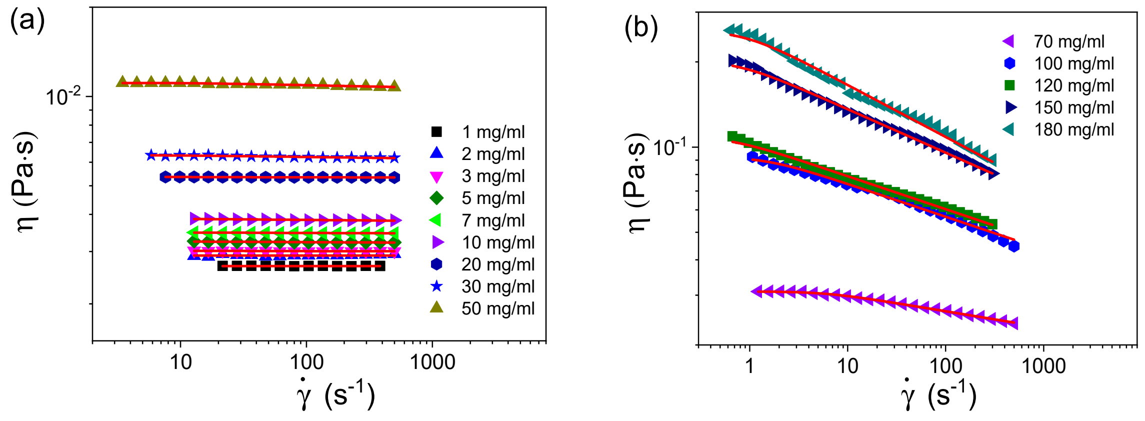

Figure S10. Dependence of viscosity on shear rate for the S-PFSA dispersions in water/ethanol with concentrations of (a) 1-50 mg/ml and (b) 70-180 mg/ml. These viscosity data were acquired by macrorheological measurements based on steady shear.

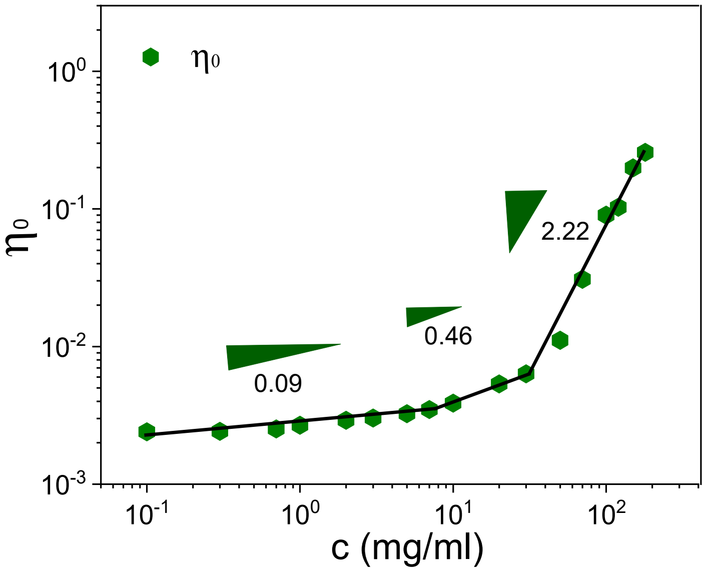

Figure S11. Dependence of zero-shear viscosity (η_0) on concentration for the S-PFSA dispersions in water/ethanol with concentrations of 1-180 mg/ml.

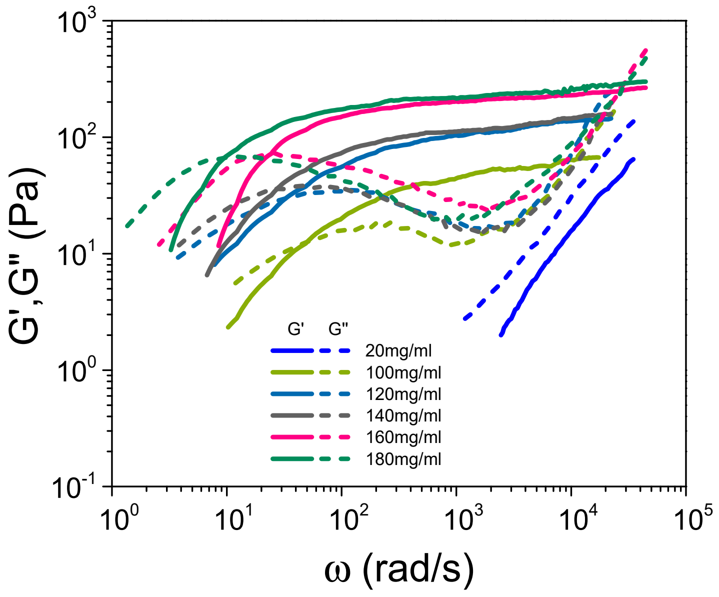

Figure S12. Frequency dependence of G′ and G″ for the S-PFSA dispersion in water/ethanol.

Table S1 The parameters for cylindrical model fitting (Eq 1,2)

c(mg/ml)	d (nm)	D(nm)	L_{cyl}
10	24	1.06	9.774
20	20.1	0.96	11.992
30	14.8	0.91	14.133
50	11.9	0.97	14.772
100	8.1	0.93	18.53
200	6.1	1.13	20.317

Table S2 The parameters for multiscale structure fitting (Eq 3, n=2)

c(mg/ml)	R_{g1} (nm)	P_1	R_{g2}(nm)	P_2
10	1.223	2.06	12.178	1.5
20	2.763	2.7	7.646	1.49
30	2.573	2.77	6.393	1.85
50	2.269	3.17	5.312	1.95
100	1.979	3.28	3.967	2.53
200	1.215	3.37	2.975	3.37

References

1. Pinder, D. N.; Swanson, A. J.; Hebraud, P.; Hemar, Y., Micro-rheological investigation of dextran solutions using diffusing wave spectroscopy. *Food Hydrocolloids* **2006,** *20* (2), 240-244.
2. Ilyin, S. O.; Malkin, A. Y.; Kulichikhin, V. G.; Denisova, Y. I.; Krentsel, L. B.; Shandryuk, G. A.; Litmanovich, A. D.; Litmanovich, E. A.; Bondarenko, G. N.; Kudryavtsev, Y. V., Effect of Chain Structure on the Rheological Properties of Vinyl Acetate–Vinyl Alcohol Copolymers in Solution and Bulk. *Macromolecules* **2014,** *47* (14), 4790-4804.
3. Mason, T. G., Estimating the viscoelastic moduli of complex fluids using the generalized Stokes–Einstein equation. *Rheol. Acta* **2000,** *39* (4), 371-378.
4. Mason, T. G.; Ganesan, K.; van Zanten, J. H.; Wirtz, D.; Kuo, S. C., Particle Tracking Microrheology of Complex Fluids. *Phys. Rev. Lett.* **1997,** *79* (17), 3282-3285.
5. Mason, T. G.; Weitz, D. A., Optical Measurements of Frequency-Dependent Linear Viscoelastic Moduli of Complex Fluids. *Phys. Rev. Lett.* **1995,** *74* (7), 1250-1253.
6. Xie, H.-R.; Ji, C.-H.; Xue, S.-M.; Xu, Z.-L.; Yang, H.; Ma, X.-H., Enhanced pervaporation performance of SA-PFSA/ceramic hybrid membranes for ethanol dehydration. *Sep. Purif. Technol.* **2018,** *206*, 218-225.
7. Fang, X.; Shen, P. K.; Song, S.; Stergiopoulos, V.; Tsiakaras, P., Degradation of perfluorinated sulfonic acid films: An in-situ infrared spectro-electrochemical study. *Polym. Degradation Stab.* **2009,** *94* (10), 1707-1713.
